# microRNA Regulation of Peritoneal Cavity Homeostasis in Peritoneal Dialysis

**DOI:** 10.1155/2015/929806

**Published:** 2015-10-01

**Authors:** Melisa Lopez-Anton, Timothy Bowen, Robert H. Jenkins

**Affiliations:** Department of Nephrology, School of Medicine, College of Biomedical & Life Sciences, Cardiff University, Heath Park Campus, Cardiff CF14 4XN, UK

## Abstract

Preservation of peritoneal cavity homeostasis and peritoneal membrane function is critical for long-term peritoneal dialysis (PD) treatment. Several microRNAs (miRNAs) have been implicated in the regulation of key molecular pathways driving peritoneal membrane alterations leading to PD failure. miRNAs regulate the expression of the majority of protein coding genes in the human genome, thereby affecting most biochemical pathways implicated in cellular homeostasis. In this review, we report published findings on miRNAs and PD therapy, with emphasis on evidence for changes in peritoneal miRNA expression during long-term PD treatment. Recent work indicates that PD effluent- (PDE-) derived cells change their miRNA expression throughout the course of PD therapy, contributing to the loss of peritoneal cavity homeostasis and peritoneal membrane function. Changes in miRNA expression profiles will alter regulation of key molecular pathways, with the potential to cause profound effects on peritoneal cavity homeostasis during PD treatment. However, research to date has mainly adopted a literature-based miRNA-candidate methodology drawing conclusions from modest numbers of patient-derived samples. Therefore, the study of miRNA expression during PD therapy remains a promising field of research to understand the mechanisms involved in basic peritoneal cell homeostasis and PD failure.

## 1. Introduction

**Figure 1:**
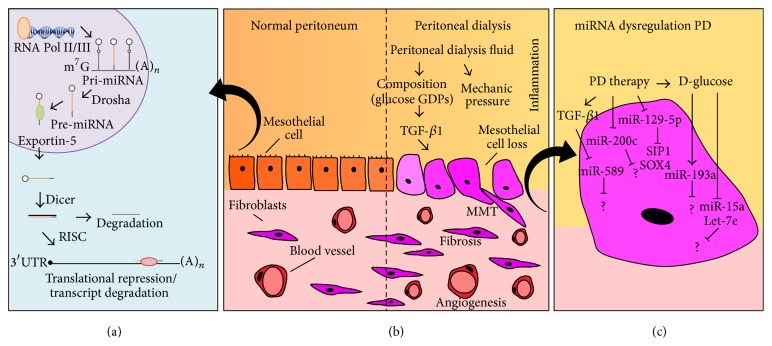
miRNA dysregulation in peritoneal dialysis. (a) miRNA biogenesis pathway. miRNAs are transcribed by RNA polymerase (Pol II) or Pol III as primary miRNA (pri-miRNA) transcripts that are processed by Drosha to generate precursor miRNAs (pre-miRNAs). Pre-miRNA hairpins are transported by Exportin-5 to the cytoplasm, where mature miRNAs are generated by Dicer, and incorporated into the RNA-induced silencing complex (RISC). miRNA-RISC complexes bind to the 3′ untranslated regions (3′ UTRs) of target mRNAs by partial complementarity, resulting in repression of translation and/or mRNA degradation. (b) Peritoneal mesothelial-to-mesenchymal transition (MMT) is associated with PD therapy. Healthy peritoneal mesothelial cells (PMCs; left hand side) undergo morphological changes during PD-driven MMT, invading the submesothelium where they contribute to angiogenesis and fibrosis and increase extracellular matrix (ECM) components deposition during PD therapy (right hand side). (c) Dysregulated miRNA expression resulting from PD therapy. Only miRNAs for which specific evidence in HPMCs exists are shown.

Peritoneal dialysis (PD) therapy involves constant exposure of the peritoneal membrane to bioincompatible PD solutions and a high basal inflammatory state. This results in an alteration of the peritoneal cavity homeostasis characterized by progressive fibrosis, angiogenesis, and ultrafiltration failure [1]. The success of long-term PD therapy depends on the maintenance of the structural and functional integrity of the peritoneal membrane, across which solute transfer occurs. Although different cell types are involved in the loss of peritoneal membrane homeostasis there is particular interest in peritoneal mesothelial cells (MCs), one of the most numerous cell types of the peritoneal cavity, 1 × 10^9^ cells. PD failure has been largely associated with the conversion of MCs to myofibroblasts, via mesothelial-to-mesenchymal transition (MMT) and mesothelial cell loss [2]. This phenotypic conversion leads to increased synthesis of extracellular matrix components and release of proinflammatory and proangiogenic factors [3] (Figure 1(b)). Therefore, PD treatment directs the fate of peritoneal homeostasis through the modulation of cell type specific signal transduction networks. The dysregulation of different molecules has been observed to play a causative role in the etiology of PD therapy. Accordingly, the determination of the upstream pathways that control the expression and/or activity of specific peritoneal cell types has turned into an important field of research.

microRNAs (miRNAs) were initially discovered in* C. elegans* as critical developmental regulators over a decade ago [4, 5]. Alterations in miRNA expression have been described in a wide range of* in vitro* and* in vivo* disease models [6]. miRNAs are short noncoding RNAs that regulate gene expression at the posttranscriptional level. Broadly, miRNAs are transcribed by RNA polymerase (Pol II) or Pol III enzymes [7, 8] as long, polyadenylated primary miRNA (pri-miRNA) molecules [9]. The pri-miRNA transcripts are processed by Drosha, a nuclear RNase III endonuclease, generating precursor miRNAs (pre-miRNA) [10]. Pre-miRNAs are 60–70 nt stem-loop hairpin molecules that are transported to the cytoplasm by Exportin-5 [11, 12]. Mature miRNAs (22–25 nt) are generated by Dicer, a cytoplasmic RNase III, and incorporated into the RNA-induced silencing complex (RISC) [13]. miRNA-RISC complex binds to 3′ untranslated regions (3′ UTRs) of specific target genes by partial complementarity, which results in repression of translation and/or degradation of the target mRNA [14]. miRNAs control the expression of the majority of protein coding genes in the human genome, thereby affecting most biochemical pathways implicated in cellular homeostasis. Additionally, one miRNA may regulate the expression of hundreds of target mRNAs, profoundly affecting cell phenotype and function. Studies on miRNA expression in different model systems and body fluids have also emphasized their potential as therapeutic targets and disease biomarkers [15–18] (Figure 1(a)).

Aberrant miRNA levels associated with PD therapy may affect the regulation of a multitude of mRNA species resulting in significant cellular effects. In the context of PD, continuous dialysis fluid exchange allows easy access to monitor peritoneal cells and miRNA expression in PD effluent (PDE), presenting the enticing possibility of monitoring peritoneal cavity homeostasis during PD treatment. In this review, we comment on published findings describing miRNAs in PD therapy, with emphasis on evidence for changes in peritoneal miRNA expression during long-term PD treatment. Compelling data suggest that miRNAs are implicated in the regulation of key molecular pathways driving peritoneal membrane alterations leading to PD failure. Additionally, miRNAs implicated in epithelial-to-mesenchymal transition (EMT) in other contexts have been associated with MCs MMT during PD therapy [19, 20]. These results have important implications for understanding peritoneal cavity alterations associated with PD therapy. However, research to date has mainly adopted a literature-based miRNA-candidate methodology drawing conclusions from modest numbers of patient-derived samples. Therefore, the study of miRNA expression during PD therapy remains a promising field of research to understand the mechanisms involved in basic peritoneal cell homeostasis and PD failure (Figure 1(c)).

## 2. miRNA-Changes during PD Therapy

miRNAs are dysregulated in a broad range of diseases. The cellular homeostasis of the peritoneal cavity is dramatically affected during long-term peritoneal dialysis (PD) therapy [21, 22]. PD treatment induces several structural and functional changes in the peritoneal cavity including cellular percentage [21, 22] and phenotypic [23] changes that may potentially be controlled by specific miRNA expression profiles. The development of micro-sample analysis techniques, together with structural and functional similarities of human peritoneal physiology compared with mouse and rat models, allowed the development of PD* in vivo* studies [24]. The establishment of competent animal models has been decisive for* in vivo* scrutiny of PD characteristics that cannot be appraised by* in vitro* models [25].

Rat PD models allow the study of long-term PD effects while being relatively economical and easy to maintain. Models based on daily intraperitoneal injections of 4.25% dextrose PD solution showed impaired peritoneal function accompanied by morphological peritoneal changes characterized by a fibroblast-like phenotype acquisition of mesothelial cells after 4 weeks [19, 26]. Total rat peritoneal RNA from this model has been analyzed by miRNA array [19, 26]. Lin et al. found robust and significant downregulation of 8 miRNAs in the hypertonic dialysate group (miR-31, miR-93, miR-100, miR-152, miR-497, miR-192, miR-194, and miR-200b) and increased expression of miR-122 was observed in the hypertonic dialysate group compared with the saline and control groups [26]. All results were RT-qPCR confirmed [26]. When the same model was analyzed by Zhou et al. [19], peritoneal fibrotic tissues displayed upregulation in 8 miRNAs (miR-205, miR-664, miR-352, miR-146b-5p, predicted miR-160, miR-132, miR-15b, and let-7d) while 15 were downregulated (miR-335, miR-923, miR-801, miR-200a, miR-801, miR-30a, miR-193a-3p, miR-193b, miR-29b, miR-203, miR-148a, miR-709, miR-192, miR-15a, and miR-26b) [19]. Among them, only miR-192 overlapped with the miRNAs described by Lin et al. [19, 26]. The authors found miR-30a downregulation particularly interesting as it is known to target EMT-related genes, such as Snail and vimentin [19, 27, 28]. Using RT-qPCR, Zhou et al. [19] validated miR-30a downregulation in total peritoneum from the rat PD model, in PD patients undergoing therapy for 3.5–66 months and following addition of TGF-*β*1 to rat primary peritoneal mesothelial cells and to human peritoneal mesothelial cell line HMrSV5 [19]. miR-30a downregulation was associated with Snail upregulation in HMrSV5 cells, in which miR-30a stable overexpression blocked TGF-*β*1-induced Snail expression resulting in inhibition of EMT [19].

A rat EMT model based on repeated exposure to glucose degradation products (GDPs) during 1-2 weeks, using methylglyoxal (MGO), has been investigated by Liu et al. [29]. Total RNA from the peritoneum of rats subjected to this model was analyzed by miRNA array [29]. Liu et al. [29] found that expression of 4 miRNAs was significantly upregulated (miR-136, miR-703, miR-30b, and miR-107), while miR-653 and miR-598 were significantly downregulated. None of these findings overlapped with the miRNAs identified by Lin et al. and Zhou et al. [19, 26, 29]. All array data were confirmed by RT-qPCR analysis, with miR-30b showing the greatest increase in the PMs of rats injected with MGO [29]. Intraperitoneal injection of miR-30b chemically modified antisense RNA oligonucleotide (ASO) in week 2 counteracted MGO-induced EMT of PMCs in rats [29]. This effect was associated with bone morphogenetic protein-7 (BMP-7), a member of the transforming growth factor-*β*1 (TGF-*β*1) superfamily that negatively regulates EMT and prevents fibrosis [30, 31]. BMP-7 was significantly downregulated after 4 weeks of MGO injection and this effect was reversed by intraperitoneal miR-30b ASO injection [29]. Finally, this group demonstrated that miR-30b directly targets BMP-7 in PMs of rats, which could antagonize the effects of TGF-*β*1 [29].

Due to significant benefits such as low cost, quick turnover, simple breeding, and multiple potential genetic manipulations, mouse PD models have become increasingly popular. Liu et al. [32] studied the expression profiles of long noncoding RNA (lncRNAs), miRNAs, and mRNAs comparing total peritoneal tissue from a mouse model of peritoneal fibrosis induced by daily intraperitoneal injection of 4.25% dextrose PD fluid (PDF) or saline solution for 4 weeks [32]. Array data showed that 14 miRNAs were upregulated and 1 miRNA was downregulated compared to normal peritoneal tissue [32]. Subsequent RT-qPCR validated upregulated expression of miR-182, miR-488, miR-292, and miR-296, while miR-200a was downregulated in the model group compared to controls [32]. Despite use of integrative pathway and coexpression network analyses, the mechanisms and functions of these miRNAs remain unclear [32].

Continuous dialysis fluid exchange offers the possibility to assess the integrity of the peritoneal membrane and characterize the functionality of the cellular components derived from PDE of patients [33, 34]. Analysis of miRNA expression profiles in total PDE cells from patients having undergone PD therapy for less than 6 months versus long-term PD patients identified downregulation of miR-129-5p, a potent downstream inhibitor of TGF-*β*1 in renal fibrosis [33]. The authors confirmed miR-129-5p downregulation by RT-qPCR and northern blot analysis and found that miR-129-5p modulated E-cadherin and vimentin expression by targeting SIP1 and SOX4 3′UTRs and modulating E-cadherin and vimentin promoter activity via the TGF-*β*1/SIP1 pathway [33]. These data suggest that miR-129-5p protects MCs undergoing MMT transformation induced by TGF-*β*1 during PD through direct targeting of SIP1 and SOX4 [33]. By contrast, Zhang et al. [34] used their unpublished data of miRNA expression profiles in HPMCs of PD patients and HMrSV5 cells treated with TGF-*β*1 to focus their studies on miR-589 [34]. miR-589 downregulation was confirmed in HPMCs from PD patients and HMrSV5 cells treated with TGF-*β*1, in which overexpression of miR-589 attenuated the EMT changes induced by TGF-*β*1 [34].

Most PD-related miRNA studies have taken a literature-based approach to the identification of candidate miRNAs for further analysis [20, 35–37]. The miR-29 family is known to be a potent downstream inhibitor of TGF-*β*1/Smad3 in heart, liver, lung, and kidney fibrosis [38–41]. Yu et al. [20] examined the therapeutic potential of miR-29b in a mouse model of PD induced fibrosis by daily infusion of 4.25% dextrose solution by miR-29b delivery before and at day 14 of therapy [20]. miR-29b overexpression showed a protective effect on peritoneal fibrosis including EMT and not only prevented peritoneal dysfunction when delivered before starting the therapy, but also altered the progression of the fibrosis when delivered after fibrosis establishment (day 14) [20]. Although there are several mechanisms by which miR-29b might inhibit peritoneal fibrosis, the authors focused on the transcription factor specificity protein 1 (Sp1), which is a putative target of miR-29b that plays an important role in TGF-*β*1/Smad3 pathway and may be a mechanism by which miR-29b inhibited peritoneal fibrosis [20, 42, 43].

The miR-200 family of miRNAs has been closely associated with a variety of fibrotic diseases including lung and kidney fibrosis [44, 45]. Zhang et al. [35] showed miRNA-200c downregulation when comparing PDE-derived MCs from patients that had recently started PD therapy with those undergoing PD for more than 6 months [35]. miR-200c expression also correlated with morphological changes in HPMCs suggesting that it may be associated with the EMT process [35]. Chen et al. [36] selected the following candidate miRNAs based on a report on EMT and kidney disease [46]: miR-15a, miR-17-92, miR-21, miR-30, miR-192, miR-216a, miR-217, and miR-377 [36]. Total PDE-derived cells from 110 PD patients (82 new, 28 prevalent) showed significant miRNA upregulation of miR-15a, miR-21, and miR-192 when comparing new, prevalent and UF groups, while miR-17, miR-30, and miR-377 expression was similar between groups [36]. miR-30 significantly correlated with GFR and no detectable expression of miR-216a and miR-217 was found in patient samples [36].

Bao et al. [37] studied a set of miRNAs related to kidney development and diseases (miR-193a, miR-21, miR-15a, miR-16, and let-7e) in a model of high-glucose EMT in HPMCs and found miR-193 upregulation, miR-15a and let-7e downregulation, and no significant changes for miR-16 and miR-21 [37]. miR-193a increase correlated with stimulus duration, suggesting to the authors that miR-193a may play an important role in the EMT of the PMCs and regulate peritoneal fibrosis [37].

## 3. Relevance of miRNA-Mediated Regulation of Peritoneal Cell Maintenance and Characteristics during PD Therapy

Several risk factors for PD therapy failure and/or the development of peritoneal fibrosis in PD patients have been described including biocompatibility of PD solutions, repeat peritonitis, and elevated expression of growth factors [26]. There is a continuous need to improve and promote large and well-documented multinational and multicenter PD registries which would allow research of appropriately sized cohort studies like the Global Fluid Study (GFS) and the balANZ study to overcome control group limitations [47, 48].

Animal models offer an accurate replication of PD therapy but may also increase the complexity of the study [24, 25]. The use of intraperitoneal injection of dialysate as PD model has many advantages including low cost, high practicality, and low infection risk [19, 20, 26, 29, 32]. Nevertheless, better PD models, in which a catheter is permanently inserted into the peritoneal cavity of the animals, have been already described with a minimum risk of developing exit site infection [25]. It is also important to remember that miRNA sequences are not always conserved between humans and animals [49], and analyzing their relevance in humans is, therefore, highly important (Table 1, [26, 29, 32]).

Several articles have based their miRNA microarray analysis on total peritoneal RNA samples (Table 1 [19, 26, 29, 32]). Nevertheless, it is well accepted that PD treatment induces several structural and functional changes in the peritoneal cavity including changes in the percentages of constituent cell types and phenotype [22]. miRNA expression profiles will change with each defined cell phenotype and context. Therefore, the study of miRNAs from total peritoneal samples has important associated challenges defining the specific cell type contribution and validating the changes in different, cell-specific models where mechanistic studies can be performed.

Indeed, CAPD patient PDE cell populations have been previously described as composed principally of macrophages (78%), followed by lymphocytes (12.3%), neutrophils (4.9%), eosinophils (2.6%), mesothelial cells (1.9%), and mast cells (0.3%) [21]. Therefore, although the study patients would be free of peritonitis, macrophages will still have an important contribution to the miRNA profile measured by total PDE-derived cells miRNA array. Consequently, miRNA arrays based on all PDE-derived cells and/or full membrane digests [19, 26, 29, 32] may not be a good model for mesothelial cell changes associated with PD therapy as suggested by some of the reviewed articles. Similarly, when the studied miRNAs are chosen from the existing literature, the choice of an appropriate model of study remains essential [20, 36, 37].* In vitro* models, although more simplistic, may be critical to understand the specific pattern of miRNA expression in response to a known stimulus in a specific cell type where mechanistic research can be pursued. Further* in vitro* research is required to elucidate specific changes in cellular miRNA expression and their downstream mechanistic events (Figure 1(c)).

Under normal conditions basal peritoneal fluid (PF) is maintained within the body to serve as a lubricant and a protective barrier between organs. The median total RNA concentration of PF was 775 *μ*g/L and 345 *μ*g/L interquartile range, and the number of detectable miRNAs was 397 [50]. When compared with other 11 body fluids (amniotic fluid, breast milk, bronchial lavage, cerebrospinal fluid, colostrum, plasma, pleural fluid, saliva, seminal fluid, tears, and urine), PF had the fifth highest RNA content and the fourth highest miRNAs content [50]. The 20 miRNAs with the highest concentrations in PF were miR-515-3p, miR-892a, miR-518e, miR-134, miR-509-5p, miR-223^*∗*^, miR-515-5p, miR-616, miR-302d, miR-873, miR-483-5p, miR-923, miR-374a, miR-598, miR-548b-3p, miR-1238, miR-92b, miR-498, miR-937, and miR-377^*∗*^, while those uniquely detected in peritoneal fluid were miR-129^*∗*^, miR-583, miR-223, miR-627, and miR-29b-1^*∗*^ [50]. Of the above, only miR-598 has been described to be downregulated by miRNA array data from total peritoneum in a rat model of MGO-induced EMT [29]. The authors measured miRNAs from PF supernatant for the first time, raising the possibility of using PDE from PD patients as a source of miRNAs that could be used as biomarkers to monitor PD therapy [50]. Of note, the measurement of miRNAs from patient PDE supernatant may be an important challenge due to the relatively short duration of the PD exchanges (4 h peritoneal equilibration test, PET) and the large fluid volume involved.

## 4. miRNAs as Biomarkers of the PD Cavity during PD Therapy

The National Institutes of Health (NIH) defines a biomarker as “*a characteristic that is objectively measured and evaluated as an indication of normal biological processes, pathogenic processes, or pharmacologic responses to a therapeutic intervention*” [51]. The prototypical biomarker must be well characterized and easily and effectively translated from basic research to routine clinical laboratories or point-of-care test. The best biomarker development practice can be achieved by the use of the Bradford Hill criteria: (A) strength, robust biomarker-outcome association; (B) consistency, persistence in different individuals, places, circumstances, and times; (C) specificity, diseases-explicit association; (D) temporality, the time course of changes in the biomarker and outcome occurring in parallel; (E) plausibility, reliable biomarker-pathogen connection; and (F) experimental evidence, biological biomarker understanding [51–53]. The evaluation of biomarker consistency is especially challenging as it requires the collection of large number of well-characterized clinical samples, and PD multinational and multicenter collections include the GFS and balANZ [47, 48].

Long-term PD therapy is characterized by the loss of the structural and functional integrity of the peritoneal membrane which leads to a progressive fibrosis, angiogenesis, and ultrafiltration failure resulting in a discontinuation of the therapy and, ultimately, transition to hemodialysis [1, 54, 55]. PET can provide therapy functional information but continuous morphological biopsy analysis is impractical. In PD patients, continuous dialysis fluid exchanges allow easy access to monitor potential peritoneal biomarkers for structural and functional peritoneal membrane changes. PDE contains several intraperitoneal and leukocyte-derived macromolecules, proteins, and RNA species, which may serve as potential biomarkers.

Cancer antigen 125 (CA-125) and interleukin-6 (IL-6) are the most highly studied PDE biomarkers. CA-125 is a high molecular weight glycoprotein and a significant body of work hypothesizes that the level of CA-125 in PDE correlates with mesothelial cell mass and a decline is indicative of mesothelial cell damage, EMT, and fibrosis [56–58]. However, recent evidence challenges this view and rather PDE CA-125 may instead reflect mesothelial cell damage, death, and detachment [59, 60]. Therefore, further research is needed to clarify CA-125 function, regulation of expression, and secretion/shedding. Pleiotropic cytokine IL-6 has essential roles in homeostasis including glucose metabolism, hypothalamic-pituitary-adrenal axis, acute inflammation, and wound healing [61]. IL-6 has been implicated in bacterial clearance during peritonitis and development of peritoneal fibrosis [61]. Plasma IL-6 correlates with comorbidity and survival of hemodialysis and PD patients while PDE IL-6 associates with peritoneal solute transport rate (PSTR) [47, 62]. However, the potential of IL-6 as a biomarker is hindered by intra- and interindividual variability [63]. No PDE-derived biomarker is currently used in clinical routine to monitor the homeostatic maintenance of the peritoneal cavity. Further research to identify PDE-miRNAs as biomarkers may contribute to individualizing PD therapy by indicating the adequacy of switching therapy, interrogating and discriminating clinical trials competence, and guiding the development of therapy innovations.

miRNAs have shown a sound potential as biomarkers in several fields and are easy to detect in different body fluids in which they may associate with proteins, microvesicles, exosomes, or necrotic bodies [64]. PDE-derived miRNAs may be particularly suitable as biomarkers due to their specific pattern of expression, easy detection, stability, and reliability [65–68]. PD-miRNA research is in early stage but there is a particular interest regarding miRNA as biomarkers to help individualizing PD treatment. Several studies have investigated the role of specific microRNAs in the peritoneum as discussed previously (Table 1), primarily associated with mesothelial EMT. These* in vitro* and* murine* models provide some association [26, 32, 34–37] and functional data [19, 20, 29, 33], but their utility as PDE biomarkers has yet to be established. Ultimately, unbiased multicenter miRNA expression analysis of PDE samples combined with robust function data would be essential for the establishment of miRNA-biomarkers associated with PD therapy.

The development of biomarkers in complex multifactorial disease, such as PD therapy, is especially challenging. Peritoneal therapy may require multiple biomarkers to achieve the degree of accuracy needed and different biomarkers may be required to address distinct specific questions. In this respect miRNAs are convenient as biomarkers due to easy, cost-effective, multiple-detection methods that have been recently developed.

## 5. Conclusion

Collectively, these studies suggest that miRNAs are likely to be important in the regulation of mesothelial cell phenotype and homeostasis in the peritoneal cavity during PD therapy. Measurement of miRNAs in PDE may therefore be valuable in predicting the clinical course of PD patients. However, previous studies have had significant design weaknesses. To maximize the success of identifying miRNA biomarkers for PD therapy it is important to adhere to the Bradford Hill criteria, develop a thorough mechanistic understanding of the biomarker, and ensure the clinical evaluation of independent cohorts is sufficiently powered. Collaboration between basic and clinical researchers is essential to develop robust data that can be transferred into diagnostics for clinical laboratories. Further research to identify PDE-miRNAs as biomarkers may contribute to individualizing PD therapy by indicating the adequacy of switching therapy, interrogating and discriminating clinical trial competence, and guiding the development of therapeutic innovations.

## Figures and Tables

**Table 1:** miRNAs implicated in the regulation of peritoneal cavity homeostasis during peritoneal dialysis therapy.

miRNA	Study selection	Model(s)	Target(s)	Downstream signaling	References
*Downregulation*: miR-31^*∗*^, miR-93, miR-100, miR-152, miR-497^*∗*^, miR-192, miR-194, and miR-200b^*∗*^ *Upregulation*: miR-122	Microarray analysis (rat PD model, 4 weeks, total peritoneal tissue)	Rat PD model (4 weeks, total peritoneal tissue)	No	No	[26]

*Downregulation*: miR-30a	Microarray analysis (rat PD model, 4 weeks, total peritoneal tissue)	Rat PD model (4 weeks, total peritoneal tissue) HMrSV5 and primary rat PMCs TGF-*β*1 stimulated Total peritoneal tissue from PD patients miR-30a stable overexpression in HMrSV5	Snail1^&^	miR-30a acts as a negative regulator of TGF-*β*1 and induces Snail1-dependent EMT during peritoneal fibrosis	[19]

*Downregulation*: miR-653^*∗*^, miR-598^*∗*^ *Upregulation*: miR-136, miR-703^#^, miR-30b, and miR-107	Microarray analysis (rat PD model, MGO-induced EMT, 1-2 weeks, total peritoneal tissue)	Rat MGO-induced EMT PD model (1-2 weeks, total peritoneal tissue) Rat MGO-induced EMT PD model with miR-30b-ASO *Ex vivo* rat PMCs cultured *in vitro*	BMP7 (miR-30b)	BMP-7 is downregulated in rat MGO-induced EMT PD model, reverted by miR-30b-ASO, and directly targeted by miR-30b, which could antagonize TGF-*β*1 effects	[29]

*Downregulation*: miR-200a-3p *Upregulation*: miR-182-5p^*∗*^, miR-488-5p, miR-296-3p, and miR-292-5p^*∗*^	Microarray analysis (mouse PD model, 4 weeks, total peritoneal tissue)	Mouse PD model (4 weeks, total peritoneal tissue)	No	No	[32]

*Downregulation*: miR-129-5p	Microarray analysis (PDE-derived HPMCs from PD patients)	PDE-derived HPMCs from PD patients HMrSV5 TGF-*β*1 stimulated miR-129-5p overexpression and SIP1/SOX4 knockdown in HMrSV5 TGF-*β*1 stimulated	SIP1, SOX4	miR-129-5p modulates E-cadherin and vimentin expression by targeting SIP1 and SOX4 genes or by modulating the promoter activity of E-cadherin and vimentin by the TGF-*β*1/SIP1 pathway. miR-125-5p protects MCs undergoing MMT TGF-*β*1-induced during PD and may exert protective effect targeting SIP1 and SOX4	[33]

*Downregulation*: miR-589	Unpublished (preexperiment CAPD profile miRNAs)	PDE-derived HPMCs from PD patients PDE-derived HPMCs and HMrSV5 TGF-*β*1 stimulated miR-589 overexpression in HMrSV5	No	No	[34]

*Downregulation*: miR-29b	Literature-based: studies on TGF-*β*1-mediated fibrosis	Mouse PD model with miR-29b overexpression (total omentum and peritoneal tissue)	SP1^&^	Blockade of the Sp1/TGF-*β*1/Smad3 pathway may be a mechanism by which miR-29b inhibited peritoneal fibrosis	[20]

*Downregulation*: miRNA-200c	Literature-based	PDE-derived HPMCs from PD patients	No	No	[35]

*Upregulation*: miR-15, miR-21, and miR-192 *No-changes*: miR-377, miR-30, and miR-17 *No-detection*: miR-216a, miR-217	Literature-based: studies on potential EMT miRNAs	PDE-derived cells from PD patients	No	No	[36]

*Downregulation*: miR-15a, let-7e *Upregulation*: miR-193a *No-changes*: miR-16, miR-21	Literature-based: studies related to kidney development and diseases	Cultured HPMCs stimulated by D-glucose (time course, 48 h) as a EMT model	No	No	[37]

^*∗*^miRNA sequence is not conserved between the model of study and human.

^#^miRNA sequence is not present in miRBase (v21, June 2014) for rat or human.

^&^Putative targets already described [19, 20].

## References

[1] Aroeira L. S., Aguilera A., Sánchez-Tomero J. A. (2007). Epithelial to mesenchymal transition and peritoneal membrane failure in peritoneal dialysis patients: Pathologic significance and potential therapeutic interventions. *Journal of the American Society of Nephrology*.

[2] Selgas R., Bajo A., Jiménez-Heffernan J. A. (2006). Epithelial-to-mesenchymal transition of the mesothelial cell—its role in the response of the peritoneum to dialysis. *Nephrology Dialysis Transplantation*.

[3] Loureiro J., Aguilera A., Selgas R. (2011). Blocking TGF-*β*1 protects the peritoneal membrane from dialysate-induced damage. *Journal of the American Society of Nephrology*.

[4] Wightman B., Ha I., Ruvkun G. (1993). Posttranscriptional regulation of the heterochronic gene lin-14 by lin-4 mediates temporal pattern formation in *C. elegans*. *Cell*.

[5] Lee R. C., Feinbaum R. L., Ambros V. (1993). The *C. elegans* heterochronic gene lin-4 encodes small RNAs with antisense complementarity to lin-14. *Cell*.

[6] Landgraf P., Rusu M., Sheridan R. (2007). A mammalian microRNA expression atlas based on small RNA library sequencing. *Cell*.

[7] Borchert G. M., Lanier W., Davidson B. L. (2006). RNA polymerase III transcribes human microRNAs. *Nature Structural and Molecular Biology*.

[8] Lee Y., Kim M., Han J. (2004). MicroRNA genes are transcribed by RNA polymerase II. *The EMBO Journal*.

[9] Lee Y., Jeon K., Lee J.-T., Kim S., Kim V. N. (2002). MicroRNA maturation: stepwise processing and subcellular localization. *The EMBO Journal*.

[10] Lee Y., Ahn C., Han J. (2003). The nuclear RNase III Drosha initiates microRNA processing. *Nature*.

[11] Yi R., Qin Y., Macara I. G., Cullen B. R. (2003). Exportin-5 mediates the nuclear export of pre-microRNAs and short hairpin RNAs. *Genes and Development*.

[12] Grishok A., Pasquinelli A. E., Conte D. (2001). Genes and mechanisms related to RNA interference regulate expression of the small temporal RNAs that control *C. elegans* developmental timing. *Cell*.

[13] Bernstein E., Caudy A. A., Hammond S. M., Hannon G. J. (2001). Role for a bidentate ribonuclease in the initiation step of RNA interference. *Nature*.

[14] Pasquinelli A. E. (2012). MicroRNAs and their targets: recognition, regulation and an emerging reciprocal relationship. *Nature Reviews Genetics*.

[15] Brown B. D., Naldini L. (2009). Exploiting and antagonizing microRNA regulation for therapeutic and experimental applications. *Nature Reviews Genetics*.

[16] Cortez M. A., Bueso-Ramos C., Ferdin J., Lopez-Berestein G., Sood A. K., Calin G. A. (2011). MicroRNAs in body fluids-the mix of hormones and biomarkers. *Nature Reviews Clinical Oncology*.

[17] Kota J., Chivukula R. R., O'Donnell K. A. (2009). Therapeutic microRNA delivery suppresses tumorigenesis in a murine liver cancer model. *Cell*.

[18] Turchinovich A., Weiz L., Langheinz A., Burwinkel B. (2011). Characterization of extracellular circulating microRNA. *Nucleic Acids Research*.

[19] Zhou Q., Yang M., Lan H., Yu X. (2013). MiR-30a negatively regulates TGF-*β*1-induced epithelial-mesenchymal transition and peritoneal fibrosis by targeting snai1. *American Journal of Pathology*.

[20] Yu J.-W., Duan W.-J., Huang X.-R., Meng X.-M., Yu X.-Q., Lan H.-Y. (2014). MicroRNA-29b inhibits peritoneal fibrosis in a mouse model of peritoneal dialysis. *Laboratory Investigation*.

[21] Bos H. J., Struijk D. G., Tuk C. W. (1991). Peritoneal dialysis induces a local sterile inflammatory state and the mesothelial cells in the effluent are related to the bacterial peritonitis incidence. *Nephron*.

[22] de Castro M. F., Selgas R., Jimenez C. (1994). Cell populations present in the nocturnal peritoneal effluent of patients on continuous ambulatory peritoneal dialysis and their relationship with peritoneal function and incidence of peritonitis. *Peritoneal Dialysis International*.

[23] Yáñez-Mó M., Lara-Pezzi E., Selgas R. (2003). Peritoneal dialysis and epithelial-to-mesenchymal transition of mesothelial cells. *The New England Journal of Medicine*.

[24] Lameire N., Van Biesen W., Van Landschoot M. (1998). Experimental models in peritoneal dialysis: a European experience. *Kidney International*.

[25] González-Mateo G. T., Loureiro J., Jiménez-Hefferman J. A. (2009). Chronic exposure of mouse peritoneum to peritoneal dialysis fluid: structural and functional alterations of the peritoneal membrane. *Peritoneal Dialysis International*.

[26] Lin F., Wu X., Zhang H. (2015). A microrna screen to identify regulators of peritoneal fibrosis in a rat model of peritoneal dialysis. *BMC Nephrology*.

[27] Cheng C.-W., Wang H.-W., Chang C.-W. (2012). microRNA-30a inhibits cell migration and invasion by downregulating vimentin expression and is a potential prognostic marker in breast cancer. *Breast Cancer Research and Treatment*.

[28] Kumarswamy R., Mudduluru G., Ceppi P. (2012). MicroRNA-30a inhibits epithelial-to-mesenchymal transition by targeting Snai1 and is downregulated in non-small cell lung cancer. *International Journal of Cancer*.

[29] Liu H., Zhang N., Tian D. (2014). MiR-30b is involved in methylglyoxal-induced epithelial-mesenchymal transition of peritoneal mesothelial cells in rats. *Cellular and Molecular Biology Letters*.

[30] Wang S., Chen Q., Simon T. C. (2003). Bone morphogenic protein-7 (BMP-7), a novel therapy for diabetic nephropathy. *Kidney International*.

[31] Zeisberg M., Hanai J.-I., Sugimoto H. (2003). BMP-7 counteracts TGF-*β*1-induced epithelial-to-mesenchymal transition and reverses chronic renal injury. *Nature Medicine*.

[32] Liu Y., Guo R., Hao G. (2015). The expression profiling and ontology analysis of noncoding rnas in peritoneal fibrosis induced by peritoneal dialysis fluid. *Gene*.

[33] Xiao L., Zhou X., Liu F. (2015). MicroRNA-129-5p modulates epithelial-to-mesenchymal transition by targeting SIP1 and SOX4 during peritoneal dialysis. *Laboratory Investigation*.

[34] Zhang K., Zhang H., Zhou X. (2012). miRNA589 regulates epithelial-mesenchymal transition in human peritoneal mesothelial cells. *Journal of Biomedicine and Biotechnology*.

[35] Zhang L., Liu F., Peng Y., Sun L., Chen G. (2013). Changes in expression of four molecular marker proteins and one microRNA in mesothelial cells of the peritoneal dialysate effluent fluid of peritoneal dialysis patients. *Experimental and Therapeutic Medicine*.

[36] Chen J., Kam-Tao P., Kwan B. C.-H. (2012). Relation between microRNA expression in peritoneal dialysis effluent and peritoneal transport characteristics. *Disease Markers*.

[37] Bao J. F., Hao J., Liu J., Yuan W. J., Yu Q. (2015). The abnormal expression level of microrna in epithelial-mesenchymal transition of peritoneal mesothelial cells induced by high glucose. *European Review for Medical and Pharmacological Sciences*.

[38] Zhang Y., Huang X.-R., Wei L.-H., Chung A. C., Yu C.-M., Lan H.-Y. (2014). miR-29b as a therapeutic agent for angiotensin II-induced cardiac fibrosis by targeting TGF-*β*/Smad3 signaling. *Molecular Therapy*.

[39] Roderburg C., Urban G.-W., Bettermann K. (2011). Micro-RNA profiling reveals a role for miR-29 in human and murine liver fibrosis. *Hepatology*.

[40] Cushing L., Kuang P. P., Qian J. (2011). miR-29 is a major regulator of genes associated with pulmonary fibrosis. *American Journal of Respiratory Cell and Molecular Biology*.

[41] Qin W., Chung A. C. K., Huang X. R. (2011). TGF-*β*/Smad3 signaling promotes renal fibrosis by inhibiting miR-29. *Journal of the American Society of Nephrology*.

[42] Poncelet A.-C., Schnaper H. W. (2001). Sp1 and smad proteins cooperate to mediate transforming growth factor-beta 1-induced alpha 2(i) collagen expression in human glomerular mesangial cells. *The Journal of Biological Chemistry*.

[43] Liu S., Wu L.-C., Pang J. (2010). Sp1/NF*κ*B/HDAC/*miR-29b* regulatory network in KIT-driven myeloid leukemia. *Cancer Cell*.

[44] Wang B., Koh P., Winbanks C. (2011). miR-200a prevents renal fibrogenesis through repression of TGF-*β*2 expression. *Diabetes*.

[45] Yang S., Banerjee S., de Freitas A. (2012). Participation of miR-200 in pulmonary fibrosis. *The American Journal of Pathology*.

[46] Li J. Y. Z., Yong T. Y., Michael M. Z., Gleadle J. M. (2010). Review: the role of microRNAs in kidney disease. *Nephrology*.

[47] Lambie M., Chess J., Donovan K. L. (2013). Independent effects of systemic and peritoneal inflammation on peritoneal dialysis survival. *Journal of the American Society of Nephrology*.

[48] Johnson D. W., Clarke M., Wilson V., Woods F., Brown F. G. (2010). Rationale and design of the balANZ trial: a randomised controlled trial of low GDP, neutral pH versus standard peritoneal dialysis solution for the preservation of residual renal function. *BMC Nephrology*.

[49] Kozomara A., Griffiths-Jones S. (2014). miRBase: annotating high confidence microRNAs using deep sequencing data. *Nucleic Acids Research*.

[50] Weber J. A., Baxter D. H., Zhang S. (2010). The microRNA spectrum in 12 body fluids. *Clinical Chemistry*.

[51] Biomarkers Definitions Working Group (2001). Biomarkers and surrogate endpoints: preferred definitions and conceptual framework. *Clinical Pharmacology and Therapeutics*.

[52] Aronson J. K. (2005). Biomarkers and surrogate endpoints. *British Journal of Clinical Pharmacology*.

[53] Sturgeon C., Hill R., Hortin G. L., Thompson D. (2010). Taking a new biomarker into routine use—a perspective from the routine clinical biochemistry laboratory. *Proteomics—Clinical Applications*.

[54] Williams J. D., Craig K. J., Topley N. (2002). Morphologic changes in the peritoneal membrane of patients with renal disease. *Journal of the American Society of Nephrology*.

[55] Davies S. J., Phillips L., Griffiths A. M., Russell L. H., Naish P. F., Russell G. I. (1998). What really happens to people on long-term peritoneal dialysis?. *Kidney International*.

[56] Visser C. E., Brouwer-Steenbergen J. J. E., Betjes M. G. H., Koomen G. C. M., Beelen R. H. J., Krediet R. T. (1995). Cancer antigen 125: a bulk marker for the mesothelial mass in stable peritoneal dialysis patients. *Nephrology Dialysis Transplantation*.

[57] Ho-Dac-Pannekeet M. M., Hiralall J. K., Struijk D. G., Krediet R. T. (1997). Longitudinal follow-up of CA125 in peritoneal effluent. *Kidney International*.

[58] Lopes Barreto D., Krediet R. T. (2013). Current status and practical use of effluent biomarkers in peritoneal dialysis patients. *The American Journal of Kidney Diseases*.

[59] Lai K. N., Lai K. B., Szeto C. C. (1997). Dialysate cell population and cancer antigen 125 in stable continuous ambulatory peritoneal dialysis patients: their relationship with transport parameters. *American Journal of Kidney Diseases*.

[60] Brȩborowicz A., Brȩborowicz M., Pyda M., Połubinska A., Oreopoulos D. (2005). Limitations of CA125 as an index of peritoneal mesothelial cell mass. *Nephron. Clinical Practice*.

[61] Jones S. A., Fraser D. J., Fielding C. A., Jones G. W. (2015). Interleukin-6 in renal disease and therapy. *Nephrology, Dialysis, Transplantation*.

[62] Cho Y., Johnson D. W., Vesey D. A. (2014). Dialysate interleukin-6 predicts increasing peritoneal solute transport rate in incident peritoneal dialysis patients. *BMC Nephrology*.

[63] Lopes Barreto D., Coester A. M., Noordzij M. (2011). Variability of effluent cancer antigen 125 and interleukin-6 determination in peritoneal dialysis patients. *Nephrology, Dialysis, Transplantation*.

[64] Kosaka N., Iguchi H., Ochiya T. (2010). Circulating microRNA in body fluid: a new potential biomarker for cancer diagnosis and prognosis. *Cancer Science*.

[65] Liang Y., Ridzon D., Wong L., Chen C. (2007). Characterization of microRNA expression profiles in normal human tissues. *BMC Genomics*.

[66] Lorenzen J. M., Kumarswamy R., Dangwal S., Thum T. (2012). microRNAs in diabetes and diabetes-associated complications. *RNA Biology*.

[67] Akat K. M., Moore-McGriff D., Morozov P. (2014). Comparative RNA-sequencing analysis of myocardial and circulating small RNAs in human heart failure and their utility as biomarkers. *Proceedings of the National Academy of Sciences of the United States of America*.

[68] Di Leva G., Garofalo M., Croce C. M. (2014). MicroRNAs in cancer. *Annual Review of Pathology*.

